# Disaggregating Asian American Child Welfare Involvement: Patterns, Barriers, and Policy Implications in Los Angeles County

**DOI:** 10.1177/10775595251411248

**Published:** 2026-01-05

**Authors:** Jianchao Lai, Carol Leung

**Affiliations:** 1 Department of Social Welfare, Luskin School of Public Affairs, 8783 University of California Los Angeles, Los Angeles, CA, USA; 2 School of Social Work, 14668 California State University Long Beach, Long Beach, CA, USA

**Keywords:** cultural/ethnic issues, child protective services, risk factors

## Abstract

Using administrative data from the Los Angeles County Department of Children and Family Services (DCFS) from 2017 to 2019, this study examined child welfare involvement among Asian American subgroups, addressing gaps in research through disaggregated data analysis. Findings revealed significant racial and ethnic disparities in child welfare outcomes, including differences in prior referrals, re-referrals, and case durations. While certain Asian subgroups—such as Chinese families—experienced shorter case durations, lower odds of prior referrals and recurrence compared to White families, others, including Filipino, Cambodian, Hmong, and Laotian families, showed limited or inconsistent differences from their White counterparts across these outcomes. These findings challenge the model minority stereotype, highlighting the diverse experiences of Asian American families in the child welfare system. Addressing these disparities requires disaggregated data collection and culturally responsive policies that improve service accessibility, ensure timely interventions, and better support the unique needs of Asian American subgroups.

## Introduction

Asian Americans (AAs) were the fastest-growing racial/ethnic minority group in the United States from 2000 to 2019 (Pew Research Center, 2021; U.S. Census Bureau, 2019). The AA population is projected to be the largest foreign-born immigrant group by 2055 (Pew Research Center, 2015). AAs encompass over 40 distinct subgroups with roots in East, South, and Southeast Asia, including—but not limited to—Chinese, Korean, Japanese, Indian, Pakistani, Vietnamese, Hmong, Cambodian, and Filipino communities. These subgroups vary widely in language, immigration histories, and socio-economic backgrounds (Okamoto, 2014). The AA definition was first coined in 1968 during the post-civil rights movement and has empowered this group to frame social and political identity apart from derogatory terms like “oriental “and “Asiatic” (Kibria, 1998). Similar to other pan-ethnic groups (Native Americans and Latinx), the AA construct has been categorized into a US racial system, conflating all the subgroups into one category. The lack of AA disaggregation in federal funding and data collection has perpetuated stereotypes, undermined disparities for AA, and contributed to East, South, and Southeast Asians becoming nearly synonymous with AA identity. This happens often at the expense of not recognizing distinct experiences within the Asian diaspora (Kibria, 1998; Mishra, 2016; Yoon et al., 2025).

### Unique Challenges of Asian Americans in the Child Welfare System

Although Asian Americans (AAs) are underrepresented in the child welfare system relative to other racial minority groups, they face distinct challenges. Children in AA families have patterns of risk that vary notably by nativity and ethnicity. As an example, foreign-born Asian and Pacific Islander (API) mothers, as an aggregated group, were associated with lower rates of reported maltreatment than U.S.-born API mothers (Finno-Velasquez et al., 2017). Specifically, children of foreign-born Hmong, Cambodian, Laotian, and Pacific Islander mothers had CPS report rates comparable to or higher than children of U.S.-born mothers (Finno-Velasquez et al., 2017). Moreover, parental stress in immigrant families often contributes to variations in parenting styles, including more authoritarian approaches, which may be perceived differently in the context of child welfare assessments and associated with increased maltreatment risks (Choi et al., 2020; Thor et al., 2022; Yoon et al., 2021). Similar to findings in the general population, childhood maltreatment among AAs is linked to long-term psychological consequences, such as depression, anxiety, dissociation, and suicidal ideation (Beristianos et al., 2016; Lee & Choi, 2018; Spinhoven et al., 2016). However, among AA, the emphasis on maintaining social and family harmony may lead to the internalization of these experiences, resulting in individuals suffering quietly, refraining from seeking professional help, and prioritizing “saving face” to avoid bringing shame to themselves and/or their families and preserving social reputation (Lee & Choi, 2018; Zhai & Gao, 2009).

Previous literature shows that racial disparities exist in nearly every stage of involvement in the child welfare system, including reporting, investigation, substantiation, re-referrals, and recidivism (Dettlaff & Boyd, 2020; Finno-Velasquez et al., 2017; Fluke et al., 2011). The reporting source of child welfare referrals also plays a pivotal role in shaping outcomes (Ocampo et al., 2024). Studies indicate that mandated reporters often face cultural and ethnic dilemmas when determining whether to report (Feng et al., 2012). These differential thresholds and cultural interpretations of caregiving practices can result in the over- or under-reporting of specific racial and ethnic groups, contributing to downstream disparities in service access and outcomes.

Effectively addressing racial disparities in the child welfare system requires interventions that are not only evidence-based but also culturally responsive. However, these interventions are often hindered by factors such as language barriers, parenting stress related to cultural disciplinary practices, colonialism, immigration backgrounds, and other systemic inequities that further complicate families’ experiences within the system (Finno-Velasquez et al., 2017; Huang et al., 2012). Given the diversity within the AA population and the complex sociocultural factors shaping their experiences, aggregating Asian as one racial group obscures important subgroup differences and hinder effective service provision.

#### Contextualizing Model Minority Myth

According to Kim’s (1999) theory of racial triangulation, AAs are racially positioned in ways that simultaneously uphold White dominance and marginalize their own group. They are often portrayed as “model minorities” through relative valorization, meaning they are perceived more favorably than other racial groups, while simultaneously being constructed as “foreign” and “unassimilable” through civic ostracism. The model minority myth portrays AAs as universally successful, excelling academically and economically due to cultural traits, while concealing the diversity and struggles within the community (Hsu, 2015; Walton & Truong, 2023). The chronic burden of the assumption that all AAs universally achieve higher academic, occupational, and socioeconomic statuses has perpetuated these stereotypes for over three decades (Museus & Chang, 2009; Zhou & Bankston, 2020). Although the socioeconomic diversity among AAs has been commonly shown as seeking higher education and finding higher-paying jobs, the efforts for some AAs who do not fall into this category end up having fewer resources to address educational or career opportunities and overall have social disadvantage or financial strain (Ahmmad et al., 2021; de Castro et al., 2010). The model minority myth may also serve as political propaganda for the racialization of AAs to allow for the discussion that racism did not exist in the U.S. or that racism could be overcome through hard work and abiding by American cultural norms (Hsu, 2015).

The model minority myth influences how AA parents and caregivers perceive and address racism, often through minimizing or denying its existence, which can shape their children’s understanding of racial identity (Kim et al., 2023). This myth can also overshadow the challenges faced by subgroups within the AA population and lead to the underreporting of child maltreatment. Cultural norms that emphasize family privacy, discourage outside intervention, and foster mistrust of public service systems may further discourage engagement with mandated reporters and service providers. The model minority stereotype reinforces these barriers by portraying AAs as problem-free, leading to assumptions that child maltreatment is rare within these communities. These assumptions, compounded by language barriers and a lack of culturally responsive services, can result in missed opportunities for early intervention and support (Raman & Hodes, 2012).

Meanwhile, research has shown that racial and ethnic minorities, particularly African American and Native American children, are disproportionately represented in child protective services (CPS) reports and investigations. These disparities are often attributed to a combination of systemic racism and other socio-economic factors (Kim & Drake, 2019). Systemic biases, at times, can lead to the over-surveillance of minority families and potentially exacerbate existing inequalities within the child welfare system (Dettlaff & Boyd, 2020). While AAs appear underrepresented in child welfare data, this does not necessarily reflect lower levels of risk or need. Rather, it may point to a complex interaction of underreporting, cultural stigma, and barriers to accessing services. When child welfare workers engage with AA families, the potential lack of cultural understanding or implicit biases may lead to inaccurate or ineffective interventions. For example, these workers could misinterpret ancestrally-rooted parenting practices through a Western lens, leading to incorrect interpretations around discipline, family dynamics and cultural expressions. Relying solely on aggregated AA data within child welfare systems risks masking within-group disparities and reinforcing potential gaps in service delivery.

#### Recognizing Diverse Service Needs Among AA Families

Although research on child maltreatment among disaggregated AA subgroups remains limited, existing studies emphasize the need for culturally and linguistically responsive service models, including greetings, appropriate titles, expressions of hospitality, and goal-setting practices (Hsu, 2015). These findings highlight how measurement methods can influence interpretations of disproportionality and underscore the need for culturally disaggregated data to better understand hidden disparities in service access and engagement. While some of the cultural norms and helping-seeking behaviors are similar, they are informed by distinct sociocultural lenses, historical trauma like genocide and colonialism, and migration patterns.

Within AA communities, experiences of acculturation, defined as the process of adapting to the host country, can vary widely across ethnic subgroups, immigration histories, family values, and experiences of discrimination. These factors are critical to understanding differences in help-seeking behaviors and engagement with the child welfare system. For instance, among Asian Indian families, help-seeking is often framed through a family-centered lens, with a focus on addressing collective rather than individual needs (Leung et al., 2012). Filipino Americans may be less likely to seek professional support due to the buffering effects of strong informal social networks, which can reduce perceived need for outside intervention (Martinez et al., 2020). Additionally, research showed that second-generation Korean Americans are more likely than their Filipino counterparts to retain their heritage language and reside in ethnic enclaves, which may reflect a slower or more selective acculturation process (Choi et al., 2020; Oh & Min, 2011). These variations highlight the distinct cultural contexts and lived experiences of Asian subgroups within the child welfare system.

Research on Asian American (AA) families shows that experiences in the child welfare system can vary widely by immigration history, generational background, and ethnic identity. Immigrant-origin families may encounter barriers such as limited English proficiency, social isolation, legal vulnerability, and unfamiliarity with service systems, while U.S.-born AA families more often navigate racialization, cultural stereotyping, and intergenerational conflict (Cai & Lee, 2022; Earner, 2007; Haley et al., 2022; Jang et al., 2022). These differences shape access to public assistance and child welfare services, where cultural stigma and institutional mistrust—rooted in both post-migration stressors and intergenerational trauma—can impede help-seeking (Amuedo-Dorantes & Arenas-Arroyo, 2022; Lee et al., 2010). Scholars therefore emphasize the need for culturally and linguistically responsive, trauma-informed, multiservice approaches that recognize these distinct experiences and build trust with AA families (Miller et al., 2022).

#### Asian Americans’ Childrearing Practices and Involvement With the Child Welfare System

For AA families, understanding the cultural dimensions of parenting is crucial, particularly as some immigrant parents may be unaware of U.S. definitions of child maltreatment. This lack of familiarity can lead to confusion, embarrassment or defensiveness when families are involved with the child welfare system, often through involuntary means such as mandated reports of suspected abuse or neglect (Maker et al., 2005; Son et al., 2017). In such cases, cultural understanding around discipline, supervision, or coping mechanisms may be misinterpreted. Rapid child removal, especially without cultural context and language support, can exacerbate trauma and break trust between families and the system (Trivedi, 2019). Rather than solely relying on punitive measures, the CPS has advocated providing parenting education and collaborating with families to understand disciplinary practices and systemic barriers (Terao et al., 2001).

Findings from several studies also show the differences in the reported types of child maltreatment across Asian subgroups. Significant circumstances leading up to the maltreatment show that Korean families in Los Angeles use corporal punishment (Chang et al., 2006). The study revealed Korean families are more likely to have a pattern of physical abuse (49.4%) compared to neglect (20.6%). Similarly, the most prevalent type of maltreatment among Vietnamese families was physical abuse (52%) (Rhee et al., 2012). These patterns highlight the need to move beyond pan-ethnic assumptions and attend to the specific cultural, historical, and structural contexts shaping childrearing norms across Asian subgroups. For example, corporal punishment may be perceived by some immigrant parents as a culturally appropriate form of discipline and reflect intergenerational practices (Wang & Liu, 2014; Zhai & Gao, 2009). However, these disciplinary methods may conflict with U.S. child protection standards, particularly when they result in physical injury or emotional distress.

### Research Gap and Aims

Despite the growing AA population in the U.S., research on their experiences within the child welfare system remains limited, particularly at the ethnic subgroup level. This study addresses this gap by analyzing administrative data from the Los Angeles County Department of Children and Family Services (DCFS) from 2017 to 2019 to explore racial and ethnic disparities, especially Asian subgroup variation relative to White families across three outcomes: prior involvement with child welfare system, likelihood of re-referrals, and duration of child welfare involvement. The study aims to provide critical insights for policymakers and practitioners, highlighting the need for disaggregated data and culturally tailored interventions to better support the diverse experiences and needs of AA families navigating the child welfare system.

## Methods

Data for this study was obtained from the Los Angeles County Department of Children and Family Services (LA DCFS) data management system, spanning the three-year period from 2017 to 2019. The administrative data was collected as part of the case management processes. All referrals included in the dataset were screened in and accepted for further assessment, pending substantiation or service provision. This study was approved by UCLA IRB (21-001713).

### Variables

### Dependent Variables

Three dependent variables were identified for this study. First, the re-referral count variable captured the number of times a child was referred to LA DCFS following an initial referral during the three-year study period. This variable was calculated using each child’s unique identifier to track the total number of subsequent referrals within that timeframe. The second dependent variable, the count of days referrals stayed open, was calculated as the days between the case opening date and the case close date. The last dependent variable, number of prior referrals, recorded whether any referrals occurred before the first referral for the same family during the study period.

### Independent Variables

#### Individual-Level Variables

Understanding the ethnic background of children in the child welfare system is essential for identifying potential disparities in service provision and outcomes. The child ethnicity variable represented the reported ethnicity of the child, which included racial groups (e.g., Black, White, Hispanic/Latinx, Asian, Pacific Islanders, etc.) and various Asian subgroups (e.g., Laotians, Chinese, Koreans, Hmong, Vietnamese, etc.). Individuals who identified as multiracial (e.g., Asian and another race) were excluded from the analysis to maintain clarity in subgroup comparisons. Only respondents who reported a single racial identity were retained for all racial subgroup analyses. The age at referral variable indicated the age of the child at the time of referral to LA DCFS.

#### Case-Level Variables

The dataset included several variables identifying the key characteristics of the referrals. Domestic violence, coded as a binary variable, indicated whether there was evidence of violence or conflict between adult household members at the time of referral. This may include intimate partner violence (IPV) or child exposure to adult domestic conflict, consistent with definitions used by child welfare systems such as DCFS. Similarly, substance abuse, also a binary variable, captured whether substance use issues were present in the household and noted at the time of the referral. When multiple types of alleged maltreatment are recorded for the same referral, a single primary allegation was assigned to the referral based on a standard severity hierarchy: physical abuse and sexual abuse are considered most severe, then neglect (general or severe), followed by caretaker absence/incapacity, emotional abuse, and exploitation. Based on the child welfare agency’s classification system, physical abuse involved non-accidental injury to a child, while neglect referred to the failure to meet basic needs such as food, shelter, or medical care. Severe neglect captured chronic or serious omissions in caregiving, and caretaker absence or incapacity included situations in which the caregiver was unavailable due to factors such as hospitalization or incarceration. Exploitation encompassed harmful practices carried out for the benefit of another person (e.g., sex trafficking, forced labor, or coerced criminal activity). The reporter type variable identified the source of the referral and the original 32 reporter categories were consolidated into seven subgroups: relative/caregiver, CWS-related staff, health professional, school/day-care personnel, government agencies/law enforcement, other relations, and other professionals.

#### Zipcode-Level Variables

The Economic Index of Concentration at the Extremes (ICE) variable represented the economic conditions within the child’s residential zip code. It aimed to measure the extent to which a population was concentrated at the extremes of economic advantage and disadvantage. The variable was calculated using the household income data from the American Community Survey (ACS) and ranged from −1 to +1, with +1 indicating total concentration of the population in the advantaged group and −1 indicating total concentration in the disadvantaged group. ICE scores were linked to the LA DCFS administrative dataset based on the family’s residential zip code.

### Analytical Plan

Descriptive and bivariate analyses were first performed to characterize families in this study and to investigate the variance of the covariates. A logistic regression model was used to analyze the outcome of prior referral (yes/no); a linear regression model to estimate referral duration in days; and a Poisson regression model to examine the count of re-referrals. Holm-Bonferroni method was implemented to address the issue of multiple testing (Holm, 1979). The Full Information Maximum Likelihood method (FIML) was utilized in the statistical models to account for potential missing data. Clustering method was introduced to account for the variances nested under each level. Post-estimation analyses, including chi-square test, Akaike Information Criterion (AIC), and Bayesian Information Criterion (BIC) were used to examine the model fit. All model computations and post-estimation analyses were performed using Stata MP18 (StataCorp, 2023).

## Results

### Descriptive Analysis

A total of 384,217 referrals were included in the dataset from year 2017 to 2019 across all racial groups. The descriptive statistics of both dependent and independent variables outlined in Table 1. Approximately 53.22% (*n* = 204,493) had prior referrals, while 46.78% (*n* = 179,724) had their first referral in the study period. The average duration that a referral remains open was approximately 39.21 days (SD = 25.42), with the range extending from 0 to 989 days. The recurrence of referrals averaged 2.17 occurrences per family (SD = 1.77), ranging from 1 to 31.

**Table 1.** Descriptive Statistics

	*N*/Mean	%	SD	Range
Dependent variable				
Prior referral				
Yes	204,493	53.22		
No	179,724	46.78		
Days referral remains open	39.21		25.42	(0, 989)
Recurrence (count)	2.17		1.77	(1,31)
Independent variable				
Domestic violence				
Yes	44,923	11.73		
No	338,144	88.27		
Substance abuse				
Yes	91,287	23.83		
No	291,780	76.17		
Race/Ethnicity				
American Indian/Alaskan Native	542	0.14		
Asian Indian	706	0.18		
Black	67,983	17.75		
Cambodian	679	0.18		
Chinese	2,671	0.7		
Filipino	2,719	0.71		
Guamanian	47	0.01		
Hawaiian	160	0.04		
Hispanic	221,125	57.72		
Hmong	27	0.01		
Japanese	401	0.1		
Korean	1,027	0.27		
Vietnamese	937	0.24		
Laotian	79	0.02		
Other	37,495	9.79		
Other Asian	1,248	0.33		
Other Pacific Islander	288	0.08		
Polynesian	32	0.01		
Samoan	577	0.15		
White	44,324	11.57		
Child age	8.15		5.06	(0, 18)
ICE economic index	0.03		0.19	(−1, 0.73)
Allegation type				
At risk, sibling abused	87,456	22.83		
Caretaker absence/incapacity	5,065	1.32		
Emotional abuse	62,289	16.26		
Exploitation	452	0.12		
General neglect	142,322	37.15		
Physical abuse	61,432	16.04		
Severe neglect	3,014	0.79		
Sexual abuse	21,037	5.49		
SDM risk level				
Low	48,275	15.27		
Moderate	133,378	42.2		
High	99,073	31.34		
Very high	35,365	11.19		
Reporter type				
Relative/caregiver	12,932	3.88		
CWS-related staff	24,393	7.32		
Health professional	77,020	23.12		
School/day-care personnel	78,971	23.71		
Government agencies/law enforcement	86,881	26.08		
Other relations	9,599	2.88		
Other professional	43,300	13.00		

For the independent variables, 11.73% (*n* = 44,923) of referrals involved domestic violence, while substance abuse was present in 23.83% (*n* = 91,287) of referrals. Ethnicity breakdowns showed that more than half of the referrals were related to Hispanic families (57.72%, *n* = 221,125), followed by Black (17.75%, *n* = 67,983), and White families (11.57%, n = 44,324). Asian subgroups collectively comprised a small percentage, with Chinese (0.7%, *n* = 2,671), Filipino (0.71%, *n* = 2,719), Korean (0.27%, *n* = 1,027), and Vietnamese (0.24%, *n* = 937) being the most represented. The mean age at referral was 8.15 years (SD = 5.06), ranging from 0 to 18 years. Economic conditions, as measured by the ICE economic index, had a mean of 0.03 (SD = 0.19), ranging from −1 to 0.73. Allegation types varied, with general neglect being the most frequently reported type (37.15%, *n* = 142,322), followed by “At Risk, Sibling Abused” (22.83%, *n* = 87,456), and emotional abuse (16.26%, *n* = 62,289). Sexual abuse accounted for 5.49% (*n* = 21,037) of cases, while more specific forms such as severe neglect and exploitation were relatively rare (0.79% and 0.12%, respectively). In terms of SDM (Structured Decision Making) risk level, cases were distributed as follows: 15.27% (*n* = 48,275) were categorized as low risk, 42.2% (*n* = 133,378) as moderate risk, 31.34% (*n* = 99,073) as high risk, and 11.19% (*n* = 35,365) as very high risk. These risk levels provide insight into the likelihood of future harm and guide the prioritization and allocation of resources within the child welfare system.

### Analytical Results

#### Prior Referral

The results of the GLM model with logit link highlighted several significant factors associated with the likelihood of having prior referrals (Table 2). Compared to White families, several Asian ethnic groups were significantly less likely to have prior referrals, including Chinese (OR = 0.61, 95% CI: 0.44, 0.85, *p* < .001), Filipino (OR = 0.75, 95% CI: 0.59, 0.95, *p* < .05), Korean (OR = 0.59, 95% CI: 0.41, 0.85, *p* < .05), Vietnamese (OR = 0.63, 95% CI: 0.44, 0.90, *p* < .05), and Other Asian (OR = 0.69, 95% CI: 0.50, 0.93, *p* < .05). Additionally, Hmong families showed particularly low odds of prior referrals (OR = 0.14, 95% CI: 0.02, 0.96, *p* < .001), and those racial identities categorized as “Other” were also significantly less likely to have prior referrals (OR = 0.41, 95% CI: 0.33, 0.52, *p* < .001) compared to White families. In contrast, Black families were 26% more likely to have had prior referrals compared to White families (OR = 1.26, 95% CI: 1.09, 1.45, *p* < .001).

**Table 2.** Statistical Results for Prior Referral, Case Duration, and Recurrence Outcomes

Variable	If prior referral				Case duration				Recurrence			
	OR	SE	95% CI lower	95% CI upper	Coef.	SE	95% CI lower	95% CI upper	IRR	SE	95% CI lower	95% CI upper
Domestic violence	0.79*	0.03	0.74	0.85	−2.45*	0.31	−3.47	−1.43	0.99	0.02	0.93	1.03
Substance abuse	0.58*	0.01	0.56	0.60	−1.74*	0.24	−2.52	−0.96	0.87*	0.02	0.84	0.90
Race/Ethnicity												
American Indian/Alaskan Native	1.18	0.18	0.87	1.59	−1.70	1.65	−7.02	3.61	0.92	0.14	0.69	1.24
Asian Indian	0.67	0.12	0.39	1.12	−2.19	1.57	−7.27	2.89	0.87	0.11	0.68	1.12
Black	1.26*	0.06	1.09	1.45	0.59	0.59	−1.31	2.49	1.15*	0.03	1.06	1.28
Cambodian	0.86	0.08	0.72	1.02	−3.45	1.12	−7.07	0.17	0.78*	0.05	0.65	0.94
Chinese	0.61*	0.07	0.44	0.85	−3.32*	0.81	−5.93	−0.72	0.69*	0.04	0.58	0.82
Filipino	0.75*	0.06	0.59	0.95	−1.83	0.92	−4.81	1.15	0.95	0.13	0.73	1.23
Guamanian	0.20	0.17	0.02	1.84	−0.39	3.65	−12.16	11.39				
Hawaiian	0.53	0.13	0.26	1.06	4.85	6.39	−15.79	25.48	0.62*	0.11	0.40	0.96
Hispanic	1.09	0.05	0.97	1.21	0.63	0.47	−0.89	2.16	1.06	0.02	0.99	1.12
Hmong	0.14*	0.09	0.02	0.96	19.56	9.81	−12.09	51.21	---	---	---	---
Japanese	0.65	0.13	0.37	1.12	2.96	2.86	−6.27	12.19	0.72*	0.10	0.50	0.98
Korean	0.59*	0.07	0.41	0.85	−4.73*	1.11	−8.30	−1.17	0.93	0.12	0.72	1.19
Laotian	0.38	0.15	0.13	1.23	−3.28	2.71	−12.04	5.47	0.60*	0.07	0.48	0.75
Other	0.41*	0.03	0.33	0.52	−0.17	0.49	−1.76	1.42	0.83*	0.03	0.75	0.92
Other Asian	0.69*	0.07	0.50	0.93	−1.78	1.22	−5.71	2.15	0.90	0.09	0.75	1.09
Other Pacific Islander	0.99	0.18	0.70	1.41	−0.36	1.95	−6.64	5.93	0.82	0.12	0.61	1.09
Polynesian	0.52	0.43	0.80	3.30	−0.33	3.00	−10.01	9.35	1.36	0.23	0.94	1.98
Samoan	0.96	0.24	0.60	1.56	3.35	2.90	−6.01	12.72	1.25	0.16	0.93	1.67
Vietnamese	0.63*	0.08	0.44	0.90	−1.67	1.03	−5.00	1.67	0.77*	0.06	0.62	0.97
White	(Base)	--	--	--	(Base)	--	--	--	(Base)	--	--	--
ICE economic index	0.92	0.07	0.80	1.06	4.04	2.10	−0.09	8.18	0.98	0.05	0.88	1.08
Child age	1.07*	0.00	1.06	1.07	0.09*	0.01	0.06	0.11	1.00	0.00	0.99	1.01
Allegation type												
At risk, sibling abused	1.21*	0.04	1.10	1.33	1.07*	0.28	0.18	1.96	1.03	0.03	0.94	1.34
Caretaker absence/incapacity	1.22*	0.08	1.02	1.46	−10.78*	0.62	−12.78	−8.78	1.07	0.04	0.95	1.20
Emotional abuse	1.05	0.03	0.99	1.12	2.87*	0.28	1.96	3.77	0.99	0.03	0.94	1.05
Exploitation	1.52	0.34	0.86	2.68	−13.80*	1.48	−18.58	−9.01	1.42*	0.16	1.07	1.89
General neglect	(Base)	--	--	--	(Base)	--	--	--	(Base)	--	--	--
Physical abuse	1.00	0.03	0.95	1.05	−0.51	0.22	−1.23	0.21	0.99	0.03	0.94	1.05
Severe neglect	0.78*	0.05	0.67	0.91	−5.38	0.82	−8.01	−2.75	0.88*	0.03	0.80	0.98
Sexual abuse	1.19*	0.05	1.01	1.33	0.38	0.40	−0.92	1.69	1.03	0.03	0.94	1.14
SDM score												
Low	(Base)	--	--	--	(Base)	--	--	--	(Base)	--	--	--
Moderate	5.87*	0.15	5.49	6.33	1.48*	0.25	0.68	2.28	1.22*	0.06	1.06	1.40
High	21.44*	0.97	18.73	24.53	0.63	0.32	−0.41	1.68	1.36*	0.07	1.18	1.57
Very high	41.28*	2.85	33.67	50.84	−2.80*	0.47	−4.31	−1.29	1.65*	0.08	1.43	1.89
Reporter type												
Relative/caregiver	(Base)	--	--	--	(Base)	--	--	--	(Base)	--	--	--
CWS-related staff	0.67*	0.09	0.45	0.99	−4.73*	0.50	−6.34	−3.13	0.96	0.04	0.89	1.04
Health professional	0.64*	0.03	0.56	0.73	0.32	0.38	−0.90	1.53	1.03	0.04	0.96	1.11
School/day-care personnel	0.69*	0.03	0.61	0.78	−0.16	0.40	−1.44	1.12	1.05	0.04	0.98	1.14
Government agencies/law enforcement	0.56*	0.03	0.49	0.64	0.48	0.38	−0.73	1.70	0.97	0.03	0.90	1.03
Other relations	1.02	0.05	0.93	1.14	0.92	0.64	−1.15	2.99	1.15	0.07	0.97	1.35
Other professional	0.59*	0.03	0.52	0.67	−0.96	0.42	−2.31	0.39	1.02	0.04	0.95	1.10

*Note.* * = *p* < .05 after Holm-Bonferroni Corrections for multiple testing; Hmong families were excluded from the recurrence model due to limited representation in the sample.

Across racial groups, families with issues like domestic violence (OR = 0.79, 95% CI: 0.74, 0.85, *p* < .001) or substance abuse (OR = 0.58, 95% CI: 0.56, 0.60, *p* < .001) were less likely to have had prior referrals. Child’s age was positively associated with prior referrals (OR = 1.07, 95% CI: 1.06, 1.07, *p* < .001), indicating that older children were more likely to have been referred previously while controlling for other covariates. Similarly, different types of allegations also influenced the likelihood of prior referrals. For instance, cases involving sexual abuse (OR = 1.19, 95% CI: 1.01, 1.33, *p* < 0.05), caretaker absence or incapacity (OR = 1.22, CI: 1.02, 1.46, *p* < .05), and those where a sibling was at risk (OR = 1.21, 95% CI: 1.10, 1.33, *p* < .05) were more likely to involve prior referrals. On the other hand, severe neglect (OR = 0.78, 95% CI: 0.67, 0.91, *p* < .001) was associated with a decreased likelihood. Risk level as measured by SDM scores demonstrated a graded pattern. Compared to cases rated as low risk, those rated as moderate (OR = 5.87, 95% CI: 5.49, 6.33, *p* < .001), high (OR = 21.44, 95% CI: 18.73, 24.53, *p* < .001), and very high (OR = 41.28, 95% CI: 33.67, 50.84, *p* < .001) had substantially higher odds of prior referrals. Additionally, reports made by CWS-related staff (OR = 0.67, 95% CI: 0.45, 0.99, *p* < .05), health professionals (OR = 0.64, 95% CI: 0.56, 0.73, *p* < .05), school/day-care personnel (OR = 0.69, 95% CI: 0.61, 0.78, *p* < .05), government agencies/law enforcement (OR = 0.56, 95% CI: 0.49, 0.64, *p* < .05), and other professionals (OR = 0.59, 95% CI: 0.52, 0.67, *p* < .05) were less likely to involve families with prior referrals compared to those reported by relatives or caregivers.

#### Length of Open Referral

Table 2 also presented the results from the linear regression model estimating the number of days a child welfare referral remained open. While controlling for other case characteristics, referrals involving Chinese children remained open for an average of 3.32 fewer days (Coef. = −3.32, 95% CI: −5.93, −0.72; *p* < .05), and those involving Korean children were open approximately 4.73 fewer days (Coef. = −4.73, 95% CI: −8.30, −1.17; *p* < .05) relative to cases involving White children. As for covariates, referrals presented with domestic violence allegations were associated with significantly shorter durations across racial groups, averaging 2.45 fewer days (Coef. = −2.45, 95% CI: −3.47, −1.43; *p* < .05) compared to cases without domestic violence. Similarly, cases with substance abuse allegations were open for 1.74 fewer days (Coef. = −1.74, 95% CI: −2.52, −0.96; *p* < .05). Compared to general neglect allegations, referrals with caretaker absence/incapacity remained open for 10.78 fewer days (Coef. = −10.78, 95% CI: −12.78, −8.78; *p* < .001), and those involving exploitation on average (Coef. = −13.80, 95% CI: −18.58, −9.01; *p* < .001). In contrast, emotional abuse allegations (Coef. = 2.87, 95% CI: 1.96, 3.77; *p* < .05) and cases with siblings at risk allegations (Coef. = 1.07, 95% CI: 0.18, 1.96; *p* < .05) were associated with longer referral durations, suggesting these cases may require extended time for investigation or service planning.

The Structured Decision Making (SDM) risk score showed a non-linear relationship with referral duration. Referrals classified as moderate risk remained open for 1.48 more days (Coef. = 1.48, 95% CI: 0.68, 2.28; *p* < .05) compared to those rated as low risk, while very-high-risk referrals were associated with significantly shorter durations (Coef. = −2.80, 95% CI: −4.31, −1.29; *p* < .05). Finally, differences in case duration also varied by referral sources. Referrals initiated by CWS-related staff were open for 4.73 fewer days (Coef. = −4.73, 95% CI: −6.34, −3.13; *p* < .05) compared to those reported by relatives or caregivers. No other reporter types were significantly different from the reference group.

#### Number of Total Re-Referrals

The results of the Poisson model analyzing factors associated with the recurrence of referrals were presented in Table 2. The incidence rate ratios (IRR) indicated the relative change in the rate of recurrence associated with each variable. While holding the case-specific covariates constant, differences in referral recurrence rates were observed across ethnic groups when compared to the reference group (White). Black children had a significantly higher recurrence rate (IRR = 1.15, 95% CI: 1.06, 1.28, *p* < .05), while several Asian subgroups, such as Cambodians (IRR = 0.78, 95% CI: 0.65, 0.94, *p* < .05), Chinese (IRR = 0.69, 95% CI: 0.58, 0.82, *p* < .05), Japanese (IRR = 0.72, 95% CI: 0.50, 0.98, *p* < .05), Laotians (IRR = 0.60, 95% CI: 0.48, 0.75, *p* < .05), and Vietnamese (IRR = 0.77, 95% CI: 0.62, 0.97, *p* < .05) had significantly lower recurrence rates compared to White children. Hawaiian (IRR = 0.62, 95% CI: 0.40, 0.96, *p* < .05) families and those whose racial identities were categorized as “other” (IRR = 0.83, 95% CI: 0.75, 0.92, *p* < .05) were also less likely to have re-referrals within the study period.

While holding the racial component constant, the presence of substance abuse was associated with a significantly reduced rate of recurrence (IRR = 0.87, 95% CI: 0.84, 0.90, *p* < .001). Among allegation types, exploitation-related referrals had a significantly higher recurrence rate (IRR = 1.42, 95% CI: 1.07, 1.89, *p* < .05), while children with severe neglect had a lower recurrence rate (IRR = 0.88, 95% CI: 0.80, 0.98, *p* < .05) compared to general neglect cases. Higher SDM risk levels repeatedly showed associations with increased referral recurrence. Compared to low-risk cases, moderate-risk cases had a 22% higher recurrence rate (IRR = 1.22, 95% CI: 1.06, 1.40, *p* < .05), as did high-risk (IRR = 1.36, 95% CI: 1.18, 1.57, *p* < .05) and very high-risk cases (IRR = 1.65, 95% CI: 1.43, 1.89, *p* < .05).

## Discussion

The analysis revealed notable disparities in child welfare outcomes among racial and ethnic groups, demonstrating that Asian subgroups do not share uniform advantages. Certain Asian ethnicities, such as Chinese and Vietnamese, had lower odds of prior referrals and recurrence rate than White families, while other Asian subgroups were not significantly different from their White counterparts. Notably, these lower referral rates may not necessarily indicate fewer incidents of maltreatment but could instead reflect underreporting of “domestic issues,” including domestic violence and child maltreatment, within Asian communities. Cultural norms emphasizing family privacy, concerns about “losing face,” and the stigma associated with seeking external help often discourage formal help-seeking behaviors (Wong-Padoongpatt et al., 2024). Additionally, stigma and a lack of trust in external support systems, such as substance misuse treatment centers, further hinders engagement with child welfare services.

The heterogeneity in outcomes across Asian subgroups when compared to White families also highlighted the limitations of the model minority narrative, which conceals the socioeconomic, religious, and cultural diversity within these communities (Shams, 2020; Walton & Truong, 2023). One potential explanation for the shorter referral periods observed among referrals involving Chinese and Korean children, relative to White children, may lie in the geographic concentration of these communities in metropolitan areas with more developed and culturally responsive infrastructures. Los Angeles County is home to one of the largest Korean diasporas outside of Korea, as well as long-established Chinese immigrant communities. The strong presence of community-based organizations, bilingual service providers, and culturally tailored advocacy networks in these areas may help families navigate services more efficiently and reduce barriers to initial referral, despite the broader systemic challenges that other Asian subgroups may face. On the contrary, other Asian subgroups, such as Filipinos, Laotians, and Cambodians did not consistently show significant differences from their White counterparts across the three outcomes. This finding may be partly attributable to limited sample sizes and reduced statistical power. The findings suggested that reform efforts should be attentive to ethnic variation and avoid “one-size-fits-all” strategies that risk reinforcing existing inter and intra-group disparities.

Moreover, the finding that families with domestic violence or substance abuse issues were less likely to have had prior referrals across racial and ethnic groups may reflect longstanding challenges in identifying and reporting these concerns during initial system contact. Prior research suggested that both domestic violence and substance abuse are often underreported due to stigma, fear of retaliation, or limited disclosure to mandated reporters (Jonson-Reid et al., 2007; Kohl et al., 2005). Another possibility is that these issues are independently recognized as high-risk indicators, such that their disclosure or detection prompts immediate child welfare involvement without the need for a prior pattern of referrals. In these cases, the severity or urgency of the presenting concern may lead to the case being screened in and services initiated, even in the absence of documented history, reflecting the system’s emphasis on addressing acute safety threats.

Regarding referral sources, child maltreatment reports made by caregivers or close relatives showed notable associations with elevated rates of prior referrals, possibly revealing deeper, systemic factors at the familial or community level that contribute to child welfare involvement (Rivaux et al., 2008). This pattern may suggest that family members have often hesitated to report suspected maltreatment, potentially due to concerns about family separation or cultural stigmas. Despite differences in case characteristics, referral source did not appear to significantly influence referral processing time, except for referrals made by child welfare services (CWS)-related staff, which were processed more quickly. Collectively, these findings underscore the importance of additional research examining how different referral sources influence child welfare trajectories and outcomes, which could enhance early identification mechanisms across diverse referral channels (e.g., school, medical services).

### Strengths and Limitations

This study leveraged DCFS administrative data from LA County and offers a comprehensive view of child welfare outcomes over a three-year period (2017–2019), incorporating detailed case-level information such as Asian ethnic subgroups, reporter identities, and allegation types. However, administrative data entries may be inconsistent due to varied reporting practices and the dataset used in this study does not include information on key social factors such as family sizes, immigration status, and language proficiency. The absence of these variables limits the ability to examine how assimilation or acculturation may influence child welfare outcomes. For instance, the study was unable to examine whether foreign-born versus U.S.-born families experience different referral patterns, or to assess how limited English proficiency may interact with service accessibility or referral source. Additionally, future analyses could incorporate interaction terms to explore whether the effects of risk or case-related factors—such as poverty or reporter type—vary by generational or racial status (e.g., first-generation vs. second-generation immigrant families). As a result, subgroup differences identified in this study may reflect a combination of cultural, structural, and generational dynamics that remain unobserved in our analysis.

Methodologically, the study employed clustering method to account for nested variance structures related to economic disparities. However, several limitations remained to affect the interpretation and implications of the findings. In particular, the sample sizes of several Asian subgroups were smaller compared to other racial/ethnic groups in this dataset, for which the nonsignificant findings may reflect limited sample size rather than the absence of meaningful differences. Moreover, future research should assess whether these patterns hold in other geographic contexts and datasets, including work in metropolitan areas with sizable Asian populations or through international comparisons. Similar trends have been noted in Canada, where Asian Canadian families show distinct child welfare involvement (Lee et al., 2016). Longer-term longitudinal data would also make it possible to use more advanced methods—such as time-to-event analyses—to capture temporal patterns in re-referral risk and enable deeper subgroup disaggregation and insight into culturally specific factors shaping child welfare involvement.

### Conclusion

This study provides critical insights into child welfare patterns among Asian American (AA) families, challenging the model minority stereotype by showing how specific AA subgroups differ from White families in ways that are obscured in aggregate analyses. By examining differences in prior referrals, recurrence rates, and referral processing times—each assessed relative to White families as the reference group—this study highlights the importance of data disaggregation for uncovering patterns that are otherwise obscured when Asian American (AA) families are treated as a single category. These findings advance understanding of how subgroup-specific differences emerge in relation to White families and how such distinctions shape child welfare involvement and outcomes. Disaggregated data not only improves the accuracy of identifying family needs and disparities but also supports the development of targeted, culturally responsive interventions that reflect the diverse lived experiences within AA communities. Future research can build on these findings by comparing Asian subgroups directly with one another to better capture intra-group differences among Asian American population. To translate this knowledge into practice, policymakers and practitioners could prioritize investing in community-specific supports, such as multi-service organizations and developing systems that are both data-informed and equity-driven. Future efforts to expand data infrastructure, particularly around culturally relevant indicators, will be essential to improving outcomes for diverse families and advancing more just and effective child welfare practices.

## Data Availability

The data that support the findings of this study are available from the Los Angeles County Department of Children and Family Services (DCFS), but restrictions apply to the availability of these data, which were used under license for the current study and are not publicly available.*
